# Neurochemical Effects of Chronic Administration of Calcitriol in Rats

**DOI:** 10.3390/nu6126048

**Published:** 2014-12-22

**Authors:** Pei Jiang, Li-Hong Zhang, Hua-Lin Cai, Huan-De Li, Yi-Ping Liu, Mi-Mi Tang, Rui-Li Dang, Wen-Ye Zhu, Ying Xue, Xin He

**Affiliations:** 1 Institute of Clinical Pharmacy & Pharmacology, Second Xiangya Hospital, Central South University, Changsha 410011, China; E-Mails: jpoet89@126.com (P.J.); zhanglihong0612@163.com (L.-H.Z.); ghostspecialist@163.com (H.-L.C.); yipingliu1973cn@yahoo.com.cn (Y.-P.L.); tangmimi1989@126.com (M.-M.T.); ruilidang@gmail.com (R.-L.D.); zhuwenyewawbmg@126.com (W.-Y.Z.); xueying091@126.com (Y.X.); hexin526@126.com (X.H.); 2 Department of pharmacy, Jining First People’s Hospital, Jining 272011, China

**Keywords:** vitamin D, calcitriol, neurotransmitter systems, brain function

## Abstract

Despite accumulating data showing the various neurological actions of vitamin D (VD), its effects on brain neurochemistry are still far from fully understood. To further investigate the neurochemical influence of VD, we assessed neurotransmitter systems in the brain of rats following 6-week calcitriol (1,25-dihydroxyvitamin D) administration (50 ng/kg/day or 100 ng/kg/day). Both the two doses of calcitriol enhanced VDR protein level without affecting serum calcium and phosphate status. Rats treated with calcitriol, especially with the higher dose, exhibited elevated γ-aminobutyric acid (GABA) status. Correspondingly, the mRNA expression of glutamate decarboxylase (GAD) 67 was increased. 100 ng/kg of calcitriol administration also increased glutamate and glutamine levels in the prefrontal cortex, but did not alter glutamine synthetase (GS) expression. Additionally, calcitriol treatment promoted tyrosine hydroxylase (TH) and tryptophan hydroxylase 2 (TPH2) expression without changing dopamine and serotonin status. However, the concentrations of the metabolites of dopamine and serotonin were increased and the drug use also resulted in a significant rise of monoamine oxidase A (MAO_A_) expression, which might be responsible to maintain the homeostasis of dopaminergic and serotonergic neurotransmission. Collectively, the present study firstly showed the effects of calcitriol in the major neurotransmitter systems, providing new evidence for the role of VD in brain function.

## 1. Introduction

Vitamin D (VD) is becoming increasingly recognized as a neuroactive steroid affecting brain development and function. It has been shown that VD is involved in numerous brain processes including neurotrophism, neuroimmunomodulation and neurotransmission [1]. Multiple lines of evidence suggest that VD deficiency is associated with a number of neuropsychiatric diseases including Parkinson’ disease (PD), multiple sclerosis, schizophrenia and depression. On the other hand, supplementation of VD is implicated as a promising therapeutic strategy for the prevention or the treatment of these brain-related disorders [1,2,3]. Calcitriol (1,25-dihydroxyvitamin D), the active form of VD, induces its genomic effects through its nuclear receptor, the vitamin D receptor (VDR). The ubiquitous distribution of VDR throughout the brain further strengthened the importance of VD in modulating nervous system function [4]. After binding with calcitriol, VDR dimerizes with retinoid receptor (RXR) and binds to vitamin D response elements (VDREs) to exert transcriptional control over a batch of genes [5].

VD has been shown to regulate the synthesis of a variety of neurotransmitters. Animal studies suggest that calcitriol can increase the expression of genes encoding for tyrosine hydroxylase (TH), the rate-limiting enzyme in catecholamine synthesis, and provide significant neuroprotection against the dopaminergic toxins by upregulating glial derived neurotrophic factor (GDNF) [6,7,8]. Additionally, a recent study identified the presence of VDREs on tryptophan hydroxylase (TPH) genes (which is considered as the rate-limiting step in the synthesis of the serotonin) [5]. Moreover, calcitriol may also affect the production of amino acid neurotransmitters. Altered γ-aminobutyric acid (GABA) status has been found in the brain tissues of rodents fed with a VD deficient diet [9,10], and a significant reduction of glutamate decarboxylase (GAD) 67 and GAD65 protein levels has been observed in adult VD deficient mice [10]. Likewise, the prenatal VD deficiency or developmental vitamin D (DVD) deficiency also leads to significant disturbances in neurotransmission in rats [11,12]. Although several lines of evidence point to the ability of VD in modulating neurotransmission, as far as we know, there is little available data providing direct evidence to systematically evaluate the effect of VD on neurotransmitter systems. In addition, previous studies either in VD deficient rats or animals receiving chronic calcitriol administration often resulted in a tendency to hypocalcaemia or hypercalcaemia respectively [13,14], which may potentially confound the interpretations of the findings. To further elucidate the role of VD in neurotransmitter systems, in this study, we examined the expression of the key enzymes involved in the neurotransmitter metabolism and the status of the neurotransmitters and their major metabolites in the brain of rats receiving two relative lower doses of calcitriol (50 ng/kg or 100 ng/kg), and 100 ng/kg of calcitriol was suggested as the highest nonhypercalcemic dosage [15]. Given that the pathological changes in the limbic brain regions, including the prefrontal cortex and hippocampus, are tightly linked to the neuropsychiatric disorders associated with VD deficiency [3,16], we chose to specially examine the neurochemical effects of VD in these two brain areas.

## 2. Experimental Section

### 2.1. Animals and Drug Administration

Male Sprague-Dawley rats (200–230g), 7 to 9 weeks old, were housed under standard conditions of temperature (23 ± 2 °C) and light (12:12 h light/dark cycle), with free access to food and water. Rats were fed with standard AIN93G rodent diet with 1000 IU VD/kg (custom rodent diets were purchased from Beijing HFK Bioscience, Beijing, China). All animal use procedures were carried out in accordance with the Regulations of Experimental Animal Administration issued by the State Committee of Science and Technology of the People’s Republic of China, with the approval of the Ethics Committee in our university (protocol number 037/2013). Rats were randomly allocated to one of three groups: (1) vehicle (control); (2) 50 ng/kg calcitriol; and (3) 100 ng/kg calcitriol. The animals in different groups received daily gavage of vehicle, 50 ng/kg or 100 ng/kg calcitriol between 8:00 am and 9:00 am for 6 weeks. Calcitriol (Roche, Mannheim, Germany) was suspended in saline containing 0.5% Tween 80. The animals were gently handled and the drug was administrated by an experienced researcher to minimize the unnecessary stress caused by the drug treatment procedure. All the rats were weighed every day and the doses were adjusted to the weight changes.

### 2.2. VDR Protein Analysis and Serum Biochemical Assays

To confirm the effectiveness of calcitriol administration in the brain, VDR protein level was analyzed by Western blotting. Total protein was prepared from prefrontal cortex and hippocampus, and the concentration was analyzed using Bradford method. Samples were loaded on precast 12% SDS-PAGE gels with approximately 50 µg protein in each lane. Proteins in the gels were transferred to a PVDF membrane and blocked for 1 hour in 5% non-fat dry milk in TBS-T (25 mM Tris, pH 7.5, 150 mM NaCl, 0.05% Tween-20). The VDR antibody was purchased from Santa Cruz Biotechnology Inc. (Santa Cruz, CA, USA) and diluted 1:500. All membranes were subsequently probed with a 1:4000 dilution of β-actin antibody (Proteintech, Chicago, IL, USA). The signals were normalized to β-actin as an internal standard. To rule out the influence of potentially disrupted mineral metabolism, serum calcium was measured using the *o*-cresolphthalein complexone method with a commercial kit (Sekisui Medical, Tokyo, Japan) and phosphorus was determined using molybdate UV method by an inorganic phosphorus test kit (Ningbo Medical System Biotechnology, Zhejiang, China).

### 2.3. Neurochemistry Analysis

Modified from our previously reported procedures [17], a derivative method was developed for simultaneous determination of neurotransmitters and their metabolites in rat brain homogenates using high-performance liquid chromatography coupled to tandem mass spectrometry (LC-MS/MS). Briefly, 1 mL of 85% ice-cold acetonitrile-water and 10 µL of mixed internal standard solution (containing 0.12 µg/mL 3,4-dihydroxybenzylamine, 0.19 µg/mL 5-hydroxyindole-2-carboxylic acid and 1.41 µg/mL l-aspartic acid-^13^C_4_,^15^N) were added to the brain tissues, and the mixtures were homogenized by tissue homogenizer. After vortex mixing for 5min, the mixture was centrifuged at 4 °C for 5min at 15,000 rpm. The supernatant (500 μL) was then transferred into another Eppendorf tube and subsequently evaporated to dryness under vacuum. For derivatization, 150 µL of dansyl chloride solution (4 mg/mL in acetonitrile) and 50 µL of 0.1 M Na_2_CO_3_-NaHCO_3_ buffer (pH 11.0) were added to the residue and reacted at 35 °C for 30 min. After the reaction, the pH of the mixture was adjusted to approximately 7.0 by adding 5 µL of 15% formic acid-water solution. After centrifugation at 15,000 rpm for 5 min, the supernatant was transferred to the vial and 5 μL was injected for analysis. LC-MS/MS analyses were carried out on a Waters Acquity ultra-performance liquid chromatography system (Waters, Milford, MA, USA) with a Micromass Quattro Premier XE tandem quadruple mass spectrometer (Waters, Milford, MA, USA) equipped with ESI source. The analytes were separated on an Ultimate XB-C8 column, 2.1 mm × 50 mm, 3.0 µm particle size (Welch, Shanghai, China) with the column temperature at 40 °C. The mobile phase for elution was a gradient established between solvent A (water with 20 mM ammonium acetate and 0.1% formic acid) and solvent B (acetonitrile) at a flow rate of 0.25 mL/min. The source operated in positive ion mode, and its main working parameters were set as follows: capillary voltage, 3.00 kV; extractor voltage, 3.00 V; source temperature, 120 °C; desolvation temperature, 450 °C; desolvation gas flow, 750 L/h; cone gas flow, 50 L/h. Argon used as the collision gas was introduced into the collision cell at a flow rate of 0.16 mL/min. Data acquisition was carried out by Mass Lynx 4.1 software (Waters, Manchester, UK). Neurotransmitters were quantified relative to the internal standard areas and calibrated using standard curves.

### 2.4. Real-Time PCR Analysis

Total RNA from the selected brain areas was isolated using Trizol reagent (invitrogen, Carlsbad, CA, USA) according to the manufacturer’s instructions. Quantification of mRNAs was performed on Bio-rad Cx96 Detection System (Bio-rad, Hercules, CA, USA) using SYBR green PCR kit (Applied Biosystems, Carlsbad, CA, USA) and gene-specific primers. Each cDNA was tested in triplicate with 40 cycles of amplication. Relative quantitation for PCR product was normalized to β-actin as internal standard. The sequences of gene-specific primers are summarized in Table 1.

**Table 1:** Primer sequences used for the qPCR analysis.

Gene (accession no.)	Sense Primer (5′–3′)	Antisense Primer (5′–3′)	Amplicon length
GAD65 (NM012563)	GCTCTACGGAGACTCTGAGAAG	CGGTTGGTCTGACAATTCCC	318 bp
GAD67 (NM017007)	TGTGGCGTAGCCCATGGATG	ACTGGTGTGGGTGGTGGAAG	320 bp
GS (NM017073)	CCACTGTCCCTGGGCTTAGTTTA	AGTGACATGCTAGTCCCACCAA	147 bp
TPH2 (NM173839)	GGGTTACTTTCCTCCATCGGA	AAGCAGGTTGTCTTCGGGTC	86 bp
MAO_A_ (NM033653)	GTGTGGAACCCCTTGGCATA	GTCCCATTCCTGAGCGTGTC	130 bp
IDO (NM023973)	CCAGTCCGTGAGTTTGTCATTTT	CAGTCCCTCTGTTTTCCGTGTTT	196 bp
TH (NM012740)	ACCACCTGGTCACCAAGTTT	GCAATCTCTTCCGCTGTGTA	160 bp
COMT (NM012531)	ATCTTCACGGGGTTTCAGTG	GAGCTGCTGGGGACAGTAAG	145 bp
β-Actin (NM031144)	CATCCTGCGTCTGGACCTGG	TAATGTCACGCACGATTTCC	116bp

### 2.5. Statistical Analysis

Results from the experiment were reported as means ± SEM and analyzed using SPSS version 13.0 software (SPSS Inc., Chicago, IL, USA). Differences between groups were determined by one-way ANOVA followed by Dunnett’s *t*-test for post-hoc comparisons. The prior level of significance was established at *p* < 0.05.

## 3. Results

### 3.1. VDR Expression and Serum Levels of Calcium and Phosphorus

**Figure 1:**
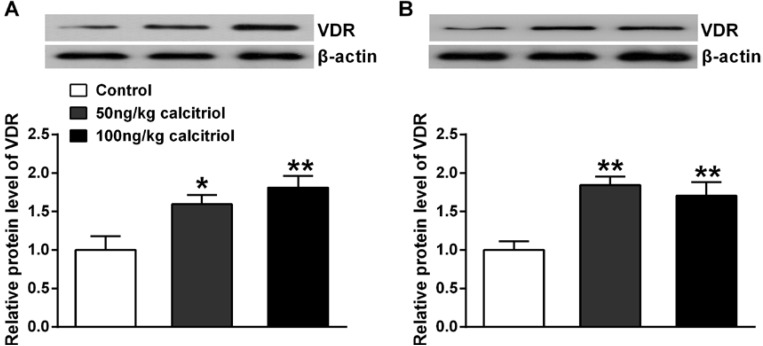
Effect of chronic calcitriol administration on protein expression of vitamin D receptor (VDR) in prefrontal cortex (**A**); and hippocampus (**B**). Relative expression values were shown as a normalized ratio to β-actin protein level. Data are means ± SEM (*n* = 4). * *p* < 0.05, ** *p* < 0.01 compared to control group.

As previous findings in adipocytes and bone cells [18,19], chronic administration of either the two doses of calcitriol also induced VDR expression in both the prefrontal cortex (Figure 1A, *p* ≤ 0.01) and hippocampus (Figure 1B, *p* ≤ 0.01). Since calcitriol can stimulate VDR expression via the activation of its gene expression and the stabilization of the receptor protein [16], our results indicate that calcitriol at these two doses both can effectively cross the blood-brain barrier and act on the brain. Additionally, both the two doses of calcitriol had no effect on body weight changes and serum levels of calcium and phosphorus (Table 2).

**Table 2:** Effect of chronic calcitriol administration on body weight gain and serum status of calcium and phosphate. Data are means ± SEM (*n* = 8).

Groups	Body weight gain (g)	Calcium (mmol/L)	Phosphate (mmol/L)
Control	162.25 ± 5.77	2.30 ± 0.06	2.59 ± 0.15
50 ng/kg calcitriol	170.02 ± 6.75	2.38 ± 0.04	2.68 ± 0.11
100 ng/kg calcitriol	161.25 ± 7.49	2.46 ± 0.05	2.56 ± 0.10

### 3.2. Brain Neurochemistry and Gene Expression

**Figure 2:**
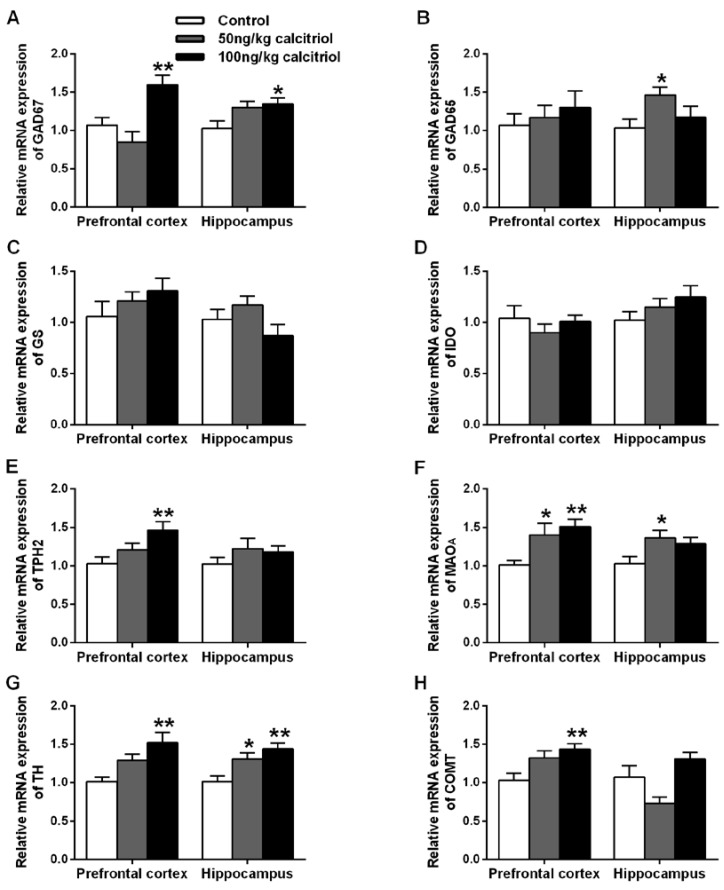
Effect of chronic calcitriol administration on mRNA levels of the metabolizing enzymes of the neurotransmitters in prefrontal cortex and hippocampus. Data are means ± SEM (*n* = 7). * *p* < 0.05, ** *p* < 0.01 compared to control group.

In the animals treated with the higher dose of calcitriol (100 ng/kg), γ-aminobutyric acid (GABA) level was significantly increased in the prefrontal cortex and hippocampus compared to the control group (Table 3). Similarly, 50 ng/kg of calcitriol also resulted in a similar trend with significant increase of GABA status in the hippocampus. Correspondingly, 100 ng/kg of calcitriol administration promoted the expression of glutamate decarboxylase (GAD) 67 in the two limbic brain regions (Figure 2A, *p* < 0.01), whereas significant increase of GAD65 expression was only found in the hippocampus of rats treated with the lower dose (Figure 2B, *p* < 0.05). 100 ng/kg of calcitriol administration also increased glutamate (Glu) (Table 3, *p* < 0.01) and glutamine (Gln) (Table 3, *p* < 0.05) levels in the prefrontal cortex, but did not alter glutamine synthetase (GS) expression (Figure 2C). Regarding the serotonergic system, calcitriol treatment had no effect on tryptophan (TRP) and serotonin (5-HT) levels and did not affect indoleamine-2,3-dioxygenase (IDO) expression (Figure 2D). Intriguingly, the expression of tryptophan hydroxylase 2 (TPH2) (Figure 2E) and monoamine oxidase A (MAO_A_) (Figure 2F) and the metabolite of 5-HT, 5-hydroxy indole acetic acid (5-HIAA), were generally enhanced, especially in the prefrontal cortex of rats treated with 100 ng/kg of calcitriol. Similarly, calcitriol (especially the higher dose) also induced tyrosine hydroxylase (TH) expression (Figure 2G) without affecting dopamine (DA) status, but led to significantly increased concentrations of DA metabolites [3,4-dihydroxyphenyl acetic acid (DOPAC) and homovanillic acid (HVA)] (Table 3) and enhanced catechol-*O*-methyltransferase (COMT) expression (Figure 2H).

**Table 3:** The content of major neurotransmitters and their metabolites in the prefrontal cortex and hippocampus of rats following 6-week administration of calcitriol. Data are means ± SEM (*n* = 7). * *p* < 0.05, ** *p* < 0.01 compared to control group.

Compound	Prefrontal cortex			Hippocampus		
	Control	50 ng/kg calcitriol	100 ng/kg calcitriol	Control	50 ng/kg calcitriol	100 ng/kg calcitriol
GABA (µg/g)	28.2 ± 2.4	31.2 ± 2.3	38.3 ± 3.1 *	20.4 ± 2.8	35.7 ± 3.2 **	31.5 ± 3.7 *
Glu (µg/g)	90.5 ± 6.5	101.8 ± 5.9	134.5 ± 6.8 **	80.1 ± 3.5	82.9 ± 10.2	99.2 ± 14.3
Gln (µg/g)	49.8 ± 3.6	56.7 ± 4.9	68.5 ± 4.5 *	38.1 ± 2.3	41.69 ± 2.69	45.90 ± 4.35
TRY (µg/g)	5.6 ± 1.0	5.5 ± 1.4	5.3 ± 0.6	5.4 ± 0.7	5.2 ± 0.4	5.3 ± 1.2
5-HT (ng/g)	919.6 ± 64.6	838.1 ± 54.3	945.7 ± 30.8	747.2 ± 32.7	833.9 ± 46.3	762.1 ± 28.1
5-HIAA (ng/g)	232.7 ± 31.6	624.1 ± 99.8 **	544.9 ± 52.7 **	343.1 ± 54.5	554.6 ± 83.6	573.5 ± 57.1 *
KYN (ng/g)	337.3 ± 24.0	284.5 ± 64.5	239.9 ± 37.1	394.4 ± 107.5	287.4 ± 82.3	416.0 ± 46.2
DA (ng/g)	551.5 ± 104.3	613.4 ± 81.0	516.7 ± 51.9	335.1 ± 26.1	399.1 ± 60.2	337.8 ± 16.7
NE (ng/g)	609.2 ± 65.4	533.5 ± 60.3	536.6 ± 29.2	546.6 ± 113.5	571.1 ±54.6	591.2 ± 64.7
DOPAC (ng/g)	115.8 ± 16.6	149.2 ± 11.9	190.9 ± 20.5 *	30.5 ± 5.1	66.1 ±7.9 **	41.7 ± 4.4
HVA (ng/g)	104.9 ± 9.5	158.9 ± 18.9 *	163.6 ± 11.4 *	83.2 ± 10.1	127.9 ± 11.0 *	178.1 ± 17.2 **

## 4. Discussion

VD has received renewed attention recently since the meteoric rise in the number of publications yielding a large amount of knowledge regarding VD and its previously unknown role in a plethora of physiological functions of the brain and associating VD deficiency with many neurological diseases. Also, treatment with VD seems likely to be a promising therapeutic strategy for the prevention or the treatment of some brain-related disorders [20,21]. It has been postulated that the alterations of neurotransmission in the key brain areas such as the prefrontal cortex and hippocampus play a pivotal role in the progression of several neuropsychiatric diseases including schizophrenia, depression and epilepsy, and the beneficial effects of VD in these brain-related disorders is, at least partially, via its modulating effect on neurotransmission [22,23]. Although several previous studies have shown some alterations in the brain of animals following either VD depletion or supplementation, these results are difficult to interpret as the hyper-/hypocalcemia-prone state may also lead to significant changes in neurotransmitter systems. For example, prolonged VD deficiency in young rats could lead to an elevation in GABA concentration in the brain but this increase was abolished following the normalization of calcium levels [14]. The impact of adult VD deficiency on brain neurochemistry and behavioral profiles in normocalcaemic animals was recently assessed [9]. However, these results might be confounded by the multitiered battery of behavioral tests (such as open field, forced swimming and hot plate test) since the various behavioral tests may cause additional stress which may result in the significant changes in the neurochemical profiles. It should be noted that there is a possibility that our results may also be affected by the potential stressful situations such as animal weighting, drug treatment and execution procedures, which might be a limitation of the study. In hope of coming nearer to address the neurochemical effects of VD, we assessed the neurotransmitter systems in rats following chronic administration of two nonhypercalcemic doses of calcitriol. Despite the relative lower doses compared to previous studies, the marked increase of VDR expression in the selected brain areas consolidated the blood brain barrier permeability of exogenous administration of calcitriol [18].

Concerning the role of VD in neurotransmission, its association with dopaminergic neurotransmission was mostly studied. Calcitriol has been shown to increase expression of GDNF and TH and enhance the evoked release of DA in rats treated with the dopaminergic neurotoxin, 6-hydroxydopamine, showing implications for PD therapy [7,8]. Consistent with previous findings, we confirmed the upregulation of TH expression following calcitriol treatment. However, the basal DA status was unchanged. Interestingly, the expression of MAO_A_ and COMT, the major catabolizing enzymes of DA, and the concentrations of DOPAC and HVA, the primary metabolites of DA, were concurrently elevated. This is probably because that the increasing synthesis of DA induced by the elevated TH expression may stimulate the expression of MAO_A_ and COMT in a negative feedback manner to maintain homeostatically dopaminergic neurotransmission, resulting in unchanged DA but increased DOPAC and HVA levels following the long time exposure to calcitriol [24]. In line with our findings, previous studies showed that eight days of treatment of calcitriol (1 μg/kg) also led to a significant increase in DOPAC levels in the striatum and accumbens without affecting tissue concentration of DA [24]. Similarly, calcitriol, especially the higher dose (100 ng/kg), also induced the mRNA level of TPH2, the rate-limiting enzyme in the biosynthesis of 5-HT specifically in the brain, without affecting 5-HT status, but significantly elevated 5-HIAA level. Although it has been identified that TPH2 gene has two distal activating VDRE sequences that are associated with transcriptional activation [5], our data firstly provide direct evidence for the inducing effect of VD on TPH2. In addition to TPH2, TRP also can be metabolized by IDO which is activated by inflammatory cytokines resulting in accelerated conversion of TRP to kynurenine (KYN) and reduced bioavailability of TRP for 5-HT production [25], whereas VD is a neuro-immunomodulator [26]. Thus, it is possible that VD may affect IDO activity via its immunomodulating effects. However, both the IDO expression and the tissue levels of TRP and KYN were unchanged, indicating that calcitriol appears to exert no effect on KYN pathway in the brain under non-pathological conditions. Since the dysfunction of serotonergic and dopaminergic neurotransmission are both implicated in mental disorders such as depression and autism, the ability of VD in modulating the monoamine neurotransmitters metabolism may provide insight into the involvement of VD in these psychiatric pathology [27]. Apart from the biogenic amines, we found that calcitriol can also affect the neurotransmission of amino acids, elevating GABA status and GAD67/65 expression. The GAD67 isoenzyme has major importance in the production of nonvesicular GABA and it is involved in synthesis of GABA for general metabolic activity. On the other hand, GAD65 is involved in regulating the vesicular pool of GABA and it responds more quickly to demands caused by GABA neuronal activity [28]. Therefore, the induction of GAD (especially GAD67) is consistent with the increase in GABA level following chronic calcitriol administration. It was recently found that the adult VD deficient mice has decreased GAD65/67 expression [9], which run parallel with the GABA increasing effect of VD shown in the present experiment. Decreased GABA status and reduced GAD65/67 levels are highly relevant to the progression of neuropsychiatric conditions such as schizophrenia, autism, depression and epilepsy, in which suboptimal status of VD was also frequently observed [29,30,31]. These clues lead to the notion that VD may have beneficial effects on these neuropsychiatric diseases through modulating GABAergic function. Additionally, we also observed a significant rise of Glu and Gln status in the prefrontal cortex after calcitriol administration. In accordance with our findings, a recent research showed decreased levels of Glu and Gln in adult VD deficient BALB/c mice [9]. There is evidence that the dysfunction of glutamate system in the prefrontal cortex is strongly associated with schizophrenia, and in DVD deficient rats, enhanced sensitivity to the N-methyl-D-aspartic acid (NMDA) receptor antagonist, MK-801-induced schizophrenia-like behaviors and reduced NMDA receptor density have been observed [32]. Although the underlying mechanisms should be further examined, clues from the previous studies and our data all point to the hypothesis that VD can modulate Glu neurotransmission which might be associated with the development of schizophrenia.

Considering the involvement of VD in neurophysiology and the lack of serious side effects of VD supplementation, the high prevalence of VD deficiency in the neuropsychiatric diseases raises the possibility that correcting the hypovitaminosis D may have potential benefits. Although we did not observe the changes in behavior and neural network which might be a limitation of the present study, our data firstly showed the wide-ranged effects of calcitriol in the major neurotransmitter systems, providing further support for the involvement of VD in the brain function.

## 5. Conclusions

Considering the involvement of VD in neurophysiology and the lack of serious side effects of VD supplementation, the high prevalence of VD deficiency in the neuropsychiatric diseases raises the possibility that correcting the hypovitaminosis D may have potential benefits. Although we did not observe the changes in behavior and neural network which might be a limitation of the present study, our data firstly showed the wide-ranged effects of calcitriol in the major neurotransmitter systems, providing further support for the involvement of VD in the brain function.
